# Supplementary material for: HDAC1/2 inhibitor therapy improves multiple organ systems in aged mice
Source: iScience. 2023 Dec 12;27(1):108681. doi: 10.1016/j.isci.2023.108681 (PMC10805681; doi:10.1016/j.isci.2023.108681)
Supplement: Document S1. Figures S1–S4 [file mmc1.pdf]

iScience, Volume *27*

Supplemental information

HDAC1/2 inhibitor therapy improves multiple organ systems in aged mice

Alessandra Tammaro, Eileen G. Daniels, Iman M. Hu, Kelly C. `t Hart, Kim Reid, Rio P. Juni, Loes M. Butter, Goutham Vasam, Rashmi Kamble, Aldo Jongejan, Richard I. Aviv, Joris J.T.H. Roelofs, Eleonora Aronica, Reinier A. Boon, Keir J. Menzies, Riekelt H. Houtkooper, and Georges E. Janssens

Ranked drug		Lifespan or aging related effect
3	sirolimus	Extends lifespan in worms/flies/mice (Johnson 2013)
3	apigenin	Suppresses inflammation (Perrot et al 2017)
4	digoxin	Extends lifespan in worms (Wang et al 2017)
4	taxifolin	Extends lifespan in worms (Benedetti 2008)
5	staurosporine	Extends lifespan in flies (Spindler et al 2012)
5	camptothecin	Topo I inhibitor induces hormesis (Zhang et al 2015)
5	genistein	Extends lifespan in worms (Lee et al 2015)
5	catechin	Extends lifespan in worms (Saul 2009)
6	tretinoin	Treatment for photo-aged skin (Kligman 1984)
6	nifedipine	Extends lifespan in rotifers (McTavish et al 1990)
6	nilotinib	In silico identified geroprotector (Ziehm 2017)
6	fludarabine	Improves healthspan in rotifers (Snell 2016)
6	tranilast	Treatment for inflammaging related diseases (Huang 2018)
6	pterostilbene	Chemically related to resveratrol (Li 2017)
6	calcitriol	Vitamin D, extends lifespan in worms (Golegaonkar et al 2015)
6	dactinomycin	In silico identified geroprotector (Barardo et al 2017)
6	estradiol	Extends lifespan in mice (Harrison et al 2014)
6	clarithromycin	Extends lifespan in rotifers (Snell 2016)

Figure S1 | Known effects of top ranked compounds, related to Figure 1. Selection of the top ranked list of drugs mimicking the most longevity interventions in the context of aging.

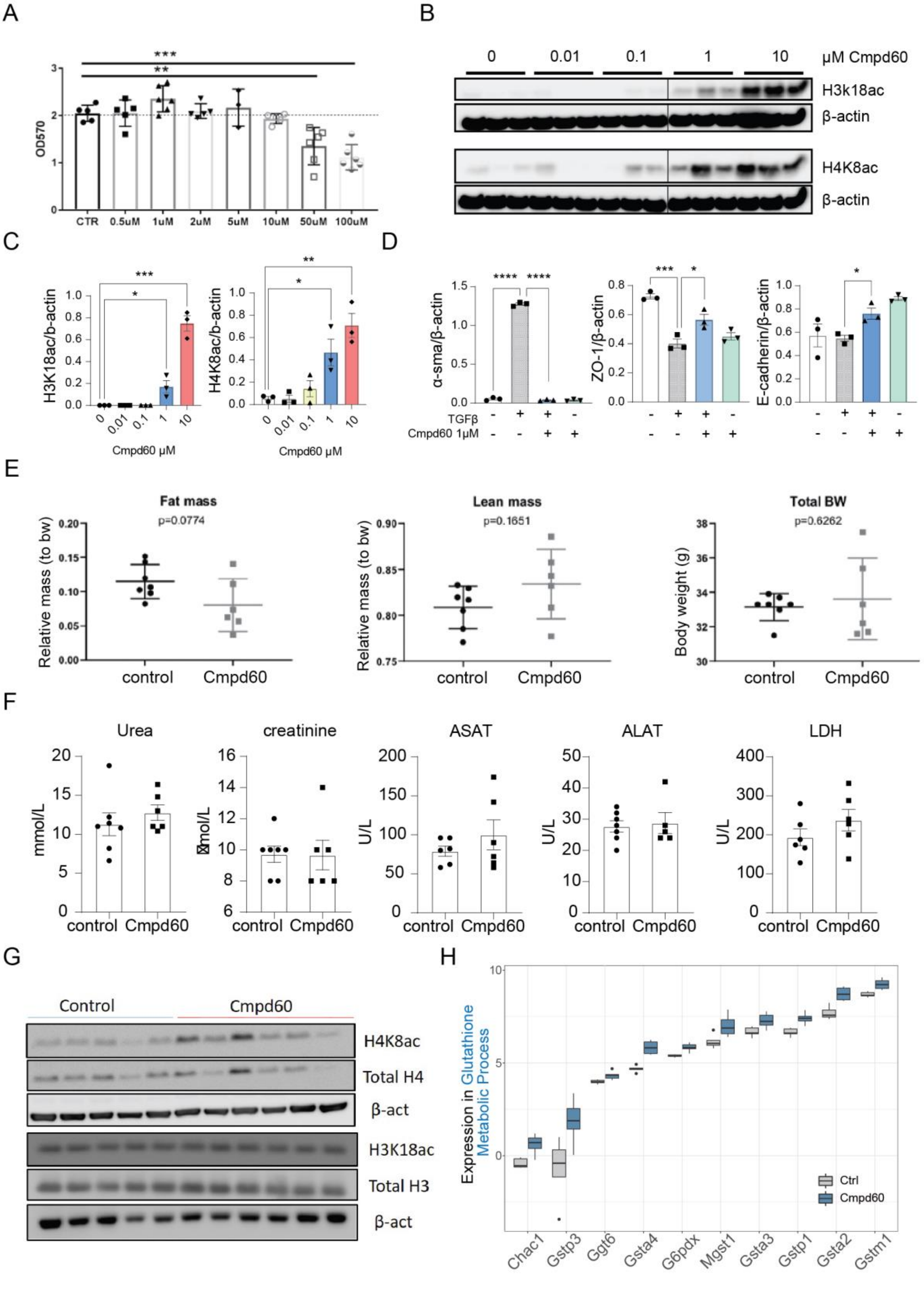

Figure S2 | Cmpd60 and aged kidney, related to Figure 2

A) MTT toxicity assay of Cmpd60 at various doses in tubular epithelial cells (TECs) stimulated for 72hours. B) Representative western blot showing expression of H3k18Ac, H4K8Ac and β-actin in TECs stimulated with different doses of Cmpd60 for 72hrs. C) Relative expression of H3k18Ac and H4K8Ac. Protein expression levels were normalized against β-actin and expressed as mean ±SEM. Three independent experiments were performed. T-test was used to determine statistical differences, *P<0.05; **P<0.01 ***P<0.001. n=3/group. D) Relative expression of α-SMA, ZO-1 and E-cadherin in TECs stimulated with TGFβ (20ng/ml) with and without 1μM Cmpd60. Protein expression levels were

normalized against β-actin and expressed as mean ±SEM. Three independent experiments were performed. Student's t-test was used to determine statistical differences, *P<0.05; ***P<0.001; ****P<0.0001 n=3/group. E) EchoMRI of aged mice treated with or without Cmpd60, shows no evidence of toxic effects (Student's t-test). F) Plasma biochemistry markers of aged mice treated with or without Cmpd60, shows no evidence of toxic effects in kidney (urea and creatinine), liver (ASAT and ALAT) and body (LDH) (Student's t-test). G) Representative western blot showing expression of H3K18ac, total H3, H4K8ac, H4 total and β-actin in aged kidneys treated with control or Cmpd60 as indicated in Figure 2B. (n = 5-6/group).

H) Boxplot of counts per million (CPM) expression values of genes in Cmpd60 treated aged mouse kidney from the GO term enrichment of glutathione metabolic process. Color represents condition, grey for control and blue for Cmpd60.

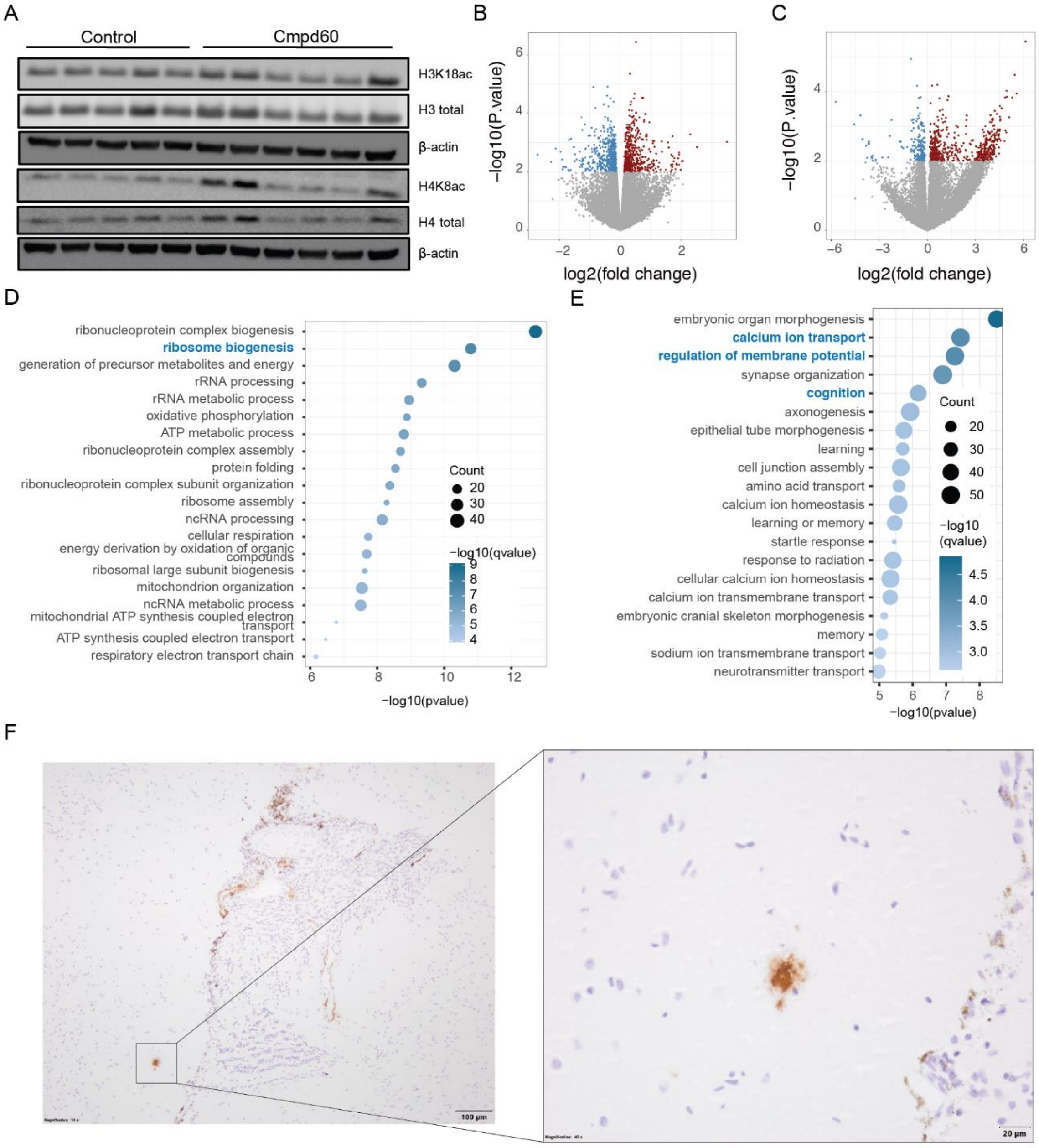

Figure S3 | Cmpd60's effects on the aged and diseased brain, related to Figure 3

A) Representative Western blot showing expression of H3K18ac, total H3, H4K8ac, H4 total and β-Actin in aged brains treated with control or Cmpd60 as indicated in Figure 2B. (n=5-6/group). B) Volcano plot of RNAseq differential expression, aged brain, treated vs untreated. Genes with p-value < 0.01 were colored (red: up-regulated, blue: down-regulated). C) Volcano plot of RNAseq differential expression, transgenic brain, treated vs untreated. Genes with p-value < 0.01 were colored (red: up-regulated, blue: down-regulated). D) GO term enrichments DOWN, transgenic brain, treated vs untreated. E) GO term enrichments UP, transgenic brain, treated vs untreated. F) Representative IHC pictures at different magnifications of β-amyloid plaques in transgenic AD mice brain.

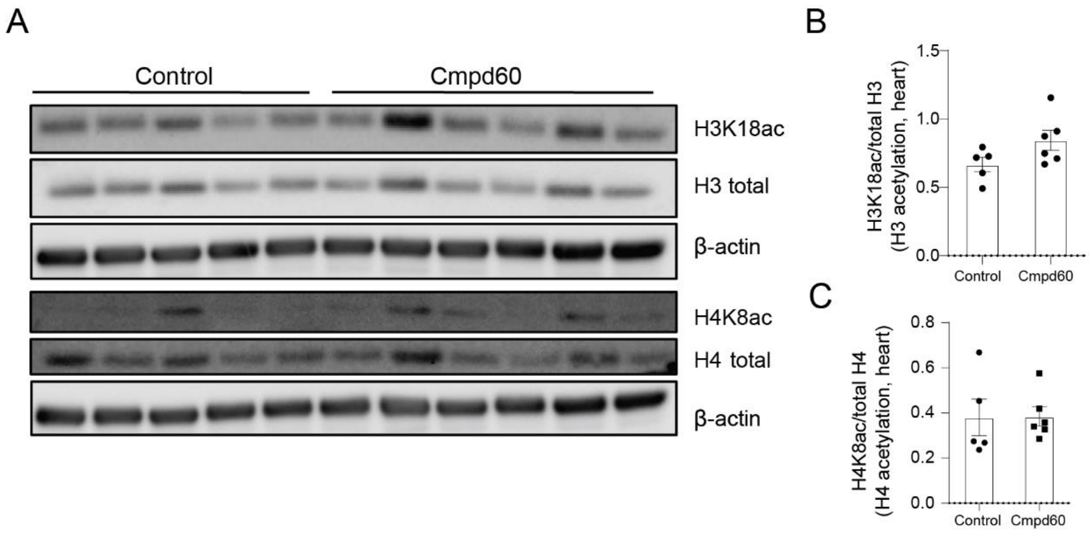

Figure S4 | The effect of Cmpd60 on heart tissue, related to Figure 4
A) Representative western blot showing expression of H3K18ac, total H3, H4K8ac, H4 total and β-Actin in aged hearts treated with control or Cmpd60 as indicated in Figure 2B (n=5-6/group). B) Relative expression of Histone H3 acetylation levels (H3k8Ac) assessed by western blot in heart tissue of aged mice treated with control and Cmpd60. Protein expression was normalized against H3 total and expressed as mean ±SEM (n=6/group). C) Relative expression of Histone H4 acetylation levels (H4k8Ac) assessed by western blot in brain tissue of aged mice treated with control and Cmpd60. Protein expression was normalized against H4 total and expressed as mean ±SEM (n=5-6/group).
